# Supplementary material for: Rootstock effects on floral induction in commercial Iranian almond cultivars: Insights from morphophysiological, biochemical, and molecular analyses
Source: PLoS One. 2025 Dec 4;20(12):e0337551. doi: 10.1371/journal.pone.0337551 (PMC12677779; doi:10.1371/journal.pone.0337551)
Supplement: S1 Table — (DOCX) [file pone.0337551.s001.docx]

| **S1 Table** | | | | |
| --- | --- | --- | --- | --- |
| **Component Weights** | **PC1** | **PC2** | **PC3** | **PC4** |
| **No. Flowers per Scaffold** | **0.290946** | **0.173796** | **-0.308114** | **0.29696** |
| **Tree Height** | **0.343255** | **0.0569827** | **-0.0384671** | **-0.363365** |
| **Scaffold length** | **0.367136** | **-0.133073** | **-0.0897492** | **0.0738821** |
| **Blooming Density** | **0.192846** | **0.227997** | **-0.318429** | **0.30481** |
| **No. Node per Scaffold** | **0.359574** | **0.155602** | **0.0218967** | **-0.122762** |
| **No. Internode per Scaffold** | **0.359574** | **0.155602** | **0.0218967** | **-0.122762** |
| **Internode Length** | **0.0991481** | **-0.445119** | **-0.134654** | **0.239856** |
| **No. lateral Branches per Scaffold** | **0.186307** | **-0.120201** | **-0.135044** | **0.581229** |
| **SCSA** | **0.283014** | **-0.191438** | **0.337392** | **0.0688316** |
| **Trunk Circumference** | **0.28349** | **-0.0355389** | **0.447764** | **0.0288958** |
| **TCSA** | **0.290152** | **-0.0712521** | **0.426566** | **0.0139728** |
| **Chl a** | **0.124234** | **0.494183** | **0.0000018176** | **0.00300739** |
| **Chl b** | **-0.179794** | **0.304269** | **0.342509** | **0.335007** |
| **Total Chl** | **-0.16181** | **0.342476** | **0.326044** | **0.316518** |
| **Carotenoids** | **0.0618612** | **0.372531** | **-0.19211** | **-0.194224** |
