# Supplementary material for: Rootstock effects on floral induction in commercial Iranian almond cultivars: Insights from morphophysiological, biochemical, and molecular analyses
Source: PLoS One. 2025 Dec 4;20(12):e0337551. doi: 10.1371/journal.pone.0337551 (PMC12677779; doi:10.1371/journal.pone.0337551)
Supplement: Attachment — Submitted filename: Response to Reviewers.docx [file pone.0337551.s005.docx]

Dear Dr. Mayank Anand Gururani,

Thank you for the opportunity to revise and resubmit our manuscript to PLOS ONE. We greatly appreciate the constructive feedback provided by you and the reviewers, which has helped us improve the quality and clarity of our manuscript. Below, we address each point raised by the academic editor and reviewers, detailing the changes made to the manuscript. We have submitted three files as requested: a rebuttal letter (this document), a revised manuscript with track changes, and a clean version of the revised manuscript.

Response to Journal Requirements

1. **PLOS ONE Style Requirements**: We have carefully reviewed and revised the manuscript to comply with PLOS ONE’s style guidelines, including file naming conventions, using the provided templates for the main body and title/authors/affiliations.

2. **Data Availability Statement**: We have included a complete Data Availability Statement in the submission form, stating: “All data are in the manuscript and supporting information files.” All relevant data have been included in the manuscript and supplementary files to ensure compliance with PLOS ONE’s data policy.

3. **Figure 1 Caption**: Figure 1 has been changed to Figure 3, and its caption has been added in line 280, describing the content and context of the figure clearly.

**Response to Reviewer Comments**

**Reviewer 1: Dr. Nimisha Sharma**

1. Abstract - 4-year study mentioned but results presented for two years

We have clarified in the abstract that the entire study, from the planting of rootstocks and grafting to the completion of evaluations, spanned four years (from April 2021 to April 2025). As described in the Materials and Methods section, the process of planting rootstocks, grafting scions onto rootstocks, and tree growth until the time of measurements and sampling took two full years (2021 to 2023). In the third year, 2024, morphological, physiological, and molecular evaluations were conducted, and these evaluations were repeated in 2025. In line 121, under the Morphological Evaluation subsection of the Materials and Methods, the sentence "In the second year (2024) after grafting the rootstocks" was revised to "In the third year (2024) after grafting the rootstocks."

2. Lack of detail for 5 bud stage and dormant stage with pictorial presentation

We have added detailed descriptions of the 5 bud stage and dormant stage in the Materials and Methods section, including their phenological characteristics. Figure 2, showing pictorial representations of these stages, has been included in the revised manuscript.

3. Provide tree and stage images in supplementary document

As suggested, we have included images of the trees and phenological stages (e.g., bud and dormant stages) in a new additional file (Figure 1) with appropriate captions.

4. Updated references missing

We have updated the reference list to include recent studies on floral induction and rootstock effects in almonds and related crops, ensuring the literature is current and relevant.

5. Current updated area and production data required

We have added a paragraph in the Introduction section citing recent global and regional almond production data (FAO) to provide context for the study’s relevance.

6. Figure quality must be improved

All figures, including the PCA figure, have been revised for improved resolution and clarity.

7. Tables’ detailed captions bulky and unclear

We have revised the captions for all tables to be concise and clear, ensuring they describe the table content without redundancy.

8. How was the hormone method optimized?

A new paragraph in the Materials and Methods section details the optimization process for hormone quantification, including calibration curves, extraction protocols, and validation steps for accuracy and reproducibility.

9. Constant gene in real-time PCR

We have specified in the Materials and Methods section that the housekeeping gene Actin (*PdActin*) was used as the reference gene for real-time PCR normalization.

10. Statistical results must be rechecked

We have rechecked all statistical analyses, including ANOVA and PCA, and confirmed their accuracy. A detailed explanation of the statistical methods, including assumptions, has been added to the Materials and Methods section.

**Reviewer 2**

1. Assumptions of ANOVA prior to analysis, especially constant variance

We have added a statement in the Materials and Methods section confirming that ANOVA assumptions, including normality (Shapiro-Wilk test) and constant variance (Levene’s test), were checked and met prior to analysis.

2. Further describe and discuss principal component analysis (PCA)

We have expanded the description of PCA in the Results section, detailing the percentage of variance explained by each principal component and the loadings of key variables. In the Discussion section, we have added a paragraph comparing our PCA findings to those in the suggested reference (Akin et al., 2022) and other relevant studies. The reference has been cited in the revised manuscript.

**Reviewer 3: Saoussen Ben Abdallah**

Abstract

1. Improve clarity and conciseness, organize results systematically

The abstract has been revised to improve clarity and conciseness, with results organized systematically (morphological, photosynthetic pigments, endogenous hormones, molecular assessments). Redundant phrases have been removed.

2. Growth regulator hormones are biochemical, not physiological parameters

We have corrected the terminology in the abstract and throughout the manuscript, referring to growth regulator hormones as biochemical parameters. The sentence "Physiological assessments included measuring the photosynthetic pigment content and growth-regulating hormones associated with floral induction" was removed from the abstract.

3. Include specific numerical values in the abstract

The abstract aims to provide a general overview of the impact of Shourab 3 rootstock and its superiority over other rootstocks, while specific numerical values and details are presented in the Results section.

4. Clarify “vegetative rootstocks” and define rootstocks

The term “vegetative rootstocks” has been clarified as “clonally propagated rootstocks” in the abstract and Introduction. A brief description of Shurab2 and Shurab3, including their origin and relevance is written in the Materials and Methods section.

5. Define abbreviations in the abstract

All abbreviations (e.g., FT, SOC1, AP1) are now defined upon first use in the abstract for clarity.

Introduction

1. More balanced review of recent literature

In the introduction, we utilized references from the past five years to ensure the use of up-to-date sources.

2. Improve flow by organizing into clear paragraphs

The Introduction has been restructured into three clear paragraphs: (1) importance of floral induction, (2) influence of rootstocks, and (3) knowledge gaps in almond research, improving the logical flow.

3. Discuss molecular mechanisms of floral induction

The molecular mechanism has been elucidated in the Discussion section.

4. Make study aims more specific

The study aims have been revised to: “the aim of this study was to evaluate the effect of five rootstocks on floral induction in two commercial almond cultivars and to identify the optimal scion-rootstock combination to enhance flower induction and flower bud formation in these cultivars.”

Materials and Methods

- Clarify experimental design

The Materials and Methods section provides a comprehensive description of the experimental design, including the number of replicates and the detailed procedure for selecting the trees..

- Include environmental conditions

The Materials and Methods section describes the environmental conditions during the 4-year study, including temperature ranges, rainfall, soil type, and irrigation practices.

- Details on molecular analyses (RT-qPCR)

The description of RT-qPCR has been expanded to include PCR conditions (e.g., cycling parameters, primer sequences), normalization method (2^-ΔΔCT), and reference gene (ACTIN). The misplaced gene expression method sentence has been moved from the Statistical Analysis section to Materials and Methods.

- Use at least three biological replicates for gene expression

Due to the economic conditions in our country and the high cost of laboratory materials, two biological replicates were selected.

- Move gene selection paragraph to Introduction

In alignment with the writing style adopted in the Materials and Methods section, this paragraph has been included within this part.

Results

-Improve clarity of figures, especially PCA

The resolution of all figures, particularly the PCA biplot figure, has been enhanced.

Discussion

In the Discussion section, in addition to utilizing recent studies on other crops for comparison with the findings of this study, we included a paragraph addressing the effect of the Shurab 3 rootstock on hormone levels and the expression of flowering-related genes in each almond cultivar.

Conclusion

- Make conclusions more concrete

The Conclusion has been revised to explicitly recommend Shurab3 as a high-flowering rootstock for almond growers, with guidance on its application in regions with similar environmental conditions.

Additional Notes

- No changes were made to the financial disclosure, as the original statement remains accurate.

- All typographical and grammatical errors noted by Reviewers have been corrected, and the manuscript has been thoroughly proofread to ensure standard English.

We believe these revisions address all concerns raised by the editor and reviewers, significantly improving the manuscript’s clarity, rigor, and scientific contribution. Please let us know if further clarification or revisions are needed.

Sincerely,

Dr. Behrouz Shiran

Professor in Molecular Genetics

Department of Plant Breeding and Biotechnology

Faculty of Agriculture

Shahrekord University

Shahrekord-IRAN

Email: beshiran45@gmail.com, Shiran@agr.sku.ac.ir
