# Supplementary material for: Rootstock effects on floral induction in commercial Iranian almond cultivars: Insights from morphophysiological, biochemical, and molecular analyses
Source: PLoS One. 2025 Dec 4;20(12):e0337551. doi: 10.1371/journal.pone.0337551 (PMC12677779; doi:10.1371/journal.pone.0337551)
Supplement: Attachment — Submitted filename: Response to Reviewers.docx [file pone.0337551.s007.docx]

**Response to Editor and Reviewers**

**Manuscript Title**: Evaluation of Rootstock Influence on Floral Induction in Commercial Almond Cultivars.
**Manuscript ID**: PONE-D-25-22239R1
**Corresponding Author**: Dr. Behrouz Shiran
**Journal**: PLOS ONE
**Date**:

**Dear Dr. Gururani and Reviewers**,

We sincerely thank you and the reviewers for your time and constructive feedback on our manuscript. We appreciate the opportunity to revise and improve our work and are grateful for the thoughtful comments that have helped us strengthen the clarity, structure, and scientific rigor of our study.

Below, we provide a detailed, point-by-point response to each of the editor’s and reviewers' comments. All changes have been incorporated into the revised manuscript, which includes both a tracked changes version and a clean version for your review. Revised figures have been improved for quality and clarity and submitted according to journal guidelines.

**Reviewer 1:**

Comment 1: Authors addressed most of the comments; still there is scope for improvement in the manuscript. Improve the language and quality of the Figures.
Response:
Thank you for the feedback. We have thoroughly revised the manuscript for clarity, grammar, and overall English language quality. Additionally, we improved the resolution and formatting of all figures, particularly Figures 4 and 7, as per your and Reviewer 3’s suggestions. Figures have been processed through the PACE tool as required by PLOS guidelines.

**Reviewer 3:**

Abstract

Comment 1: Add a brief introductory sentence to provide context and justify the study.
Response:
We have added a sentence at the beginning of the abstract:

“The selection of appropriate rootstocks is crucial for enhancing floral induction and optimizing almond yield, thereby markedly influencing orchard productivity.”

Comment 2: Define abbreviations (IAA, ABA, GA₃) on first use.
Response:
We have defined all abbreviations (indole-3-acetic acid [IAA], abscisic acid [ABA], and gibberellic acid [GA₃]) at their first mention in both the Abstract and Introduction.

Comment 3 & 4: Clarify and consistently mention all five rootstocks; they are inconsistently described in the abstract and results.
Response:
We revised the abstract to clearly name and discuss all five rootstocks (GN15, GF677, GN15-M, Shurab2, and Shurab3) and summarized key findings for each where relevant.

Comment 5: Not all rootstocks mentioned in the experimental design appear in the results.
Response:
We reviewed the results section and ensured all five rootstocks are represented with consistent data reporting. As outlined in the Materials and Methods section, morphological assessments were performed to evaluate how different rootstocks influenced flowering and floral induction in two commercial cultivars. Based on these evaluations, the rootstock-scion combinations with the highest (Shurab3-Shahrud12) and lowest (GN15-Mamaee) flowering rates were chosen for further hormonal and molecular analysis. This selection aimed to enable a detailed comparison and better understanding of the hormonal and molecular mechanisms associated with the rootstock that had the most pronounced effect on flowering.

Introduction

Comment 1: Clarify and specify the objective; include the list of rootstocks and parameters studied.
Response:
We revised the last paragraph of the Introduction

Comment 2: Reorganize the introduction to better align with the evaluated parameters.
Response:

Based on the title of the research, *Evaluation of Rootstock Influence on Floral Induction in Commercial Almond Cultivars*, the introduction primarily focuses on highlighting the general significance of rootstocks and their role in floral induction and flowering, aiming to clarify the importance of the topic for the reader and outline the research objectives. The evaluation of factors through which rootstocks influence the floral induction of scions, including morphological, biochemical, and molecular assessments, is extensively discussed in the materials and methods, results, and discussion sections. Nevertheless, the structure of the introduction was refined by adding an additional paragraph.

Materials and Methods

Comment 1: In Table 1, include GenBank accession number, gene locus, and PCR product size.
Response:
We have updated Table 1 to include the GenBank accession numbers, gene loci, and expected PCR product sizes for all primers used.

Comment 2: Introduce hormones earlier in the Introduction, not just in Materials and Methods.
Response:
We have now introduced the hormones (ABA, IAA, GA₃) earlier in the Introduction by adding an additional paragraph:

“Biochemical and physiological alterations in buds and nearby organs and tissues play a crucial role in their shift from vegetative to reproductive development [19] These alterations involve changes in hormone levels—especially abscisic acid (ABA), indole-3-acetic acid (IAA), and gibberellic acid (GA3)—both within the buds and surrounding areas. Various rootstocks can trigger different hormonal responses in the scion, significantly affecting its overall growth, particularly with respect to flowering and flower initiation, which are key factors influencing yield [20].”

Results

Comment: Figures 4 and 7 are not clear.
Response:
We have revised and enhanced the quality of Figures 4 and 7 for better clarity and readability, ensuring they are publication-ready. All figures have been verified through the PACE system.

Discussion

Comment: Improve critical analysis; less descriptive and more interpretative comparison with existing literature.
Response:
We have highlighted sentences in the discussion that interpret the results in comparison with previous studies, and additional sentences have been incorporated accordingly.

Conclusion

Comment: Strengthen the link between results and practical implications.
Response:
We have revised the conclusion to highlight practical implications, particularly how the findings can inform rootstock selection to optimize floral induction and yield in almond orchards.

**Additional Revisions (Editor Instructions)**

We reviewed and updated the reference list. No retracted articles were cited. Changes made to references are noted in the track-changed version of the manuscript.

A complete, clean version and a marked-up version of the manuscript with track changes have been submitted.

Figure files have been processed through the PACE tool and uploaded in the required format.

We hope the revised manuscript meets the standards required for publication in PLOS ONE, and we are grateful for the constructive guidance provided throughout the review process.

Please do not hesitate to contact us if any additional information is needed.

Kind regards,
Dr. Behrouz Shiran

Professor in Molecular Genetics

Department of Plant Breeding and Biotechnology

Faculty of Agriculture

Shahrekord University

Shahrekord-IRAN
