# Supplementary material for: Rootstock effects on floral induction in commercial Iranian almond cultivars: Insights from morphophysiological, biochemical, and molecular analyses
Source: PLoS One. 2025 Dec 4;20(12):e0337551. doi: 10.1371/journal.pone.0337551 (PMC12677779; doi:10.1371/journal.pone.0337551)
Supplement: Attachment — Submitted filename: Response_to_Reviewers_R4.docx [file pone.0337551.s008.docx]

**Response to Editor and Reviewers**

**Manuscript Title**: Rootstock Effects on Floral Induction in Commercial Iranian Almond Cultivars: Insights from Morphophysiological, Biochemical, and Molecular Analyses

**Manuscript ID**: PONE-D-25-22239R2
**Corresponding Author**: Dr. Behruz Shiran
**Journal**: PLOS ONE

**Dear Dr. Gururani and Reviewers**,

We sincerely thank you and the reviewers for your time and constructive feedback on our manuscript. We appreciate the opportunity to revise and improve our work and are grateful for the thoughtful comments that have helped us strengthen the clarity, structure, and scientific rigor of our study.

Below, we provide a detailed, point-by-point response to each of the editor’s and reviewers' comments. All changes have been incorporated into the revised manuscript, which includes both a tracked changes version and a clean version for your review. Revised figures have been improved for quality and clarity and submitted according to journal guidelines.

**Reviewer Comment 1:** The English language requires significant improvement. I recommend that the authors seek assistance from a native English speaker or a professional editor.
**Response:** The entire manuscript has been thoroughly revised by a native English scientific editor to improve clarity, grammar, and readability.

**Reviewer Comment 2:** Revise the title to make it more precise and impactful.
**Response:** The title has been revised accordingly. Previous title: “**Evaluation of Rootstock Influence on Floral Induction in Commercial Almond Cultivars**”*.* Revised title**:** “**Rootstock Effects on Floral Induction in Commercial Iranian Almond Cultivars: Insights from Morphophysiological, Biochemical, and Molecular Analyses**”.

**Reviewer Comment 3:** Improve figure clarity and resolution.
**Response:** All figures have been regenerated at ≥300 dpi with consistent font size and formatting. Compliance was verified using the PACE tool recommended by PLOS ONE.

**Reviewer Comment 4:** Abstract should introduce research problem more clearly; specify cultivars/rootstocks and rationale; use ‘scion grafted on rootstock’; mention cultivar effect in conclusion.
**Response:** The Abstract has been completely rewritten. It now starts with the importance of almond flowering, specifies the studied cultivars and rootstocks, justifies their selection, uses accurate terminology (“scion grafted on rootstock”), and explicitly addresses cultivar effects in the conclusion.

**Reviewer Comment 5:** Introduction should begin with almond importance and challenges, then rootstock-scion role; include background on cultivars/rootstocks; indicate prior studies; introduce flowering genes; avoid excessive dashes.
**Response:** The Introduction was reorganized to follow this logical sequence. Flowering-related genes analyzed in this work are introduced here, and punctuation has been corrected to replace excessive dashes.

**Reviewer Comment 6:** Clarify sampling stages (‘four stages’, ‘five stages’); include years with months; correct discrepancies in gene expression stages; define “scaffold”; specify control rootstock.
**Response:** All sampling stages have been clearly defined and harmonized. Years were added to all dates, discrepancies in gene expression stages corrected, “scaffold” defined as a main structural branch, and the control rootstock specified.

**Reviewer Comment 7:** Materials and Methods section title “Plant material and Experimental design and sampling” → remove “and sampling”.
**Response:** Corrected. The title now reads “Plant material and Experimental design.”

**Reviewer Comment 8:** Correct “cultivares” → “cultivars”; remove extra dot.
**Response:** Corrected.

**Reviewer Comment 9:** In Results, specify parameter names in morphological evaluation; split Table 2 into two; define abbreviations (TCSA, CV%); add explanation for statistical letters; revise figure titles; move some PCA/clustering tables to Supplementary; remove “Dynamic” from Figure 6 title.
**Response:** All requested modifications have been implemented. Morphological parameters are specified, Table 2 was split into two for clarity, abbreviations defined in footnotes, statistical notations explained, figure titles revised, PCA/clustering tables moved to Supplementary, and Figure 6 retitled.

**Reviewer Comment 10:** Discussion should expand comparisons with previous studies (e.g., Cong et al. 2023, Sun et al. 2017, Luna et al. 1990); ensure consistency with Abstract and Conclusion.
**Response:** The Discussion has been expanded to include detailed comparisons with Cong et al. (2023), Sun et al. (2017), and Luna et al. (1990), highlighting their relevance to our findings. Consistency between Abstract, Discussion, and Conclusion has been ensured, particularly regarding Shurab3 rootstock performance with Mamaee and Shahrud12 cultivars.

**Reviewer Comment 11:** Conclusion is too long and should be concise.
**Response:** The Conclusion has been rewritten to be more concise, emphasizing only the key findings and their practical implications.
